# Prognostic factors and outcomes in women with breast cancer in Slovenia in relation to step-wise implementation of organized screening

**DOI:** 10.1371/journal.pone.0278384

**Published:** 2022-11-30

**Authors:** Sonja Tomšič, Tina Žagar, Ana Mihor, Miran Mlakar, Katarina Lokar, Katja Jarm, Vesna Zadnik

**Affiliations:** 1 Epidemiology and Cancer Registry, Institute of Oncology Ljubljana, Ljubljana, Slovenia; 2 Faculty of Medicine, University of Ljubljana, Ljubljana, Slovenia; 3 Slovenian Breast Cancer Screening Programme DORA, Institute of Oncology Ljubljana, Ljubljana, Slovenia; LSU Health Sciences Center New Orleans: Louisiana State University Health Sciences Center, UNITED STATES

## Abstract

**Introduction:**

The aim of organized breast cancer screening is early detection and reduction in mortality. Organized screening should promote equal access and reduce socio-economic inequalities. In Slovenia, organized breast cancer screening achieved complete coverage in 11-years’ time. We explored whether step-wise implementation reflects in prognostic factors (earlier diagnosis and treatment) and survival of breast cancer patients in our population.

**Methods:**

Using population-based cancer registry and screening registry data on breast cancer cases from 2008–2018, we compared stage distribution and mean time to surgical treatment in (A) women who underwent at least one mammography in the organized screening programme, women who received at least one invitation but did not undergo mammography and women who did not receive any screening invitation, and in (B) women who were invited to organized screening and those who were not. We also compared net survival by stage in different groups of women according to their screening programme status.

**Results:**

Women who underwent at least one mammography in organized screening had lower disease stage at diagnosis. Time-to-treatment analysis showed mean time to surgery was shortest in women not included in organized screening (all stages = 36.0 days vs. 40.3 days in women included in organized screening). This could be due to quality assurance protocols with an obligatory multidisciplinary approach within the organized screening vs. standard treatment pathways which can vary in different (smaller) hospitals. Higher standard of care in screening is reflected in better survival in women included in organized screening (5-years net survival for regional stage: at least one mammography in the screening programme– 96%; invitation, but no mammography– 87.4%; no invitation or mammography in the screening programme– 82.6%).

**Conclusion:**

Our study, which is one of the first in central European countries, shows that introduction of organized screening has temporary effects on population cancer burden indicators already during roll-out period, which should therefore be as short as possible.

## 1 Introduction

Breast cancer is the most frequent cancer in women worldwide and also in EU-27, where the estimated incidence for 2020 was 355,457 cases, with higher burden in more developed parts [1]. In Slovenia, a central European country with a population of 2 million, breast cancer represents the second most frequent cancer in women with around one fifth of all cancer cases (closely following non-melanoma skin cancer) [2]. Compared to EU-27 Slovenian age-standardized incidence rate of breast cancer is somewhat lower (estimates for 2020 EU-27 142.8, Slovenia 121.2 per 100,000 –new European standard [3]). The incidence trends in EU-27 are also mainly increasing, whereas mortality trends tend to be in decline, as in Slovenia as in EU-27, mainly due to more effective treatment and earlier diagnosis [1, 2]. Primary prevention measures for breast cancer are scarce, but early detection of cancer, either through early diagnosis of symptomatic breast cancer or by screening asymptomatic women, is part of cancer control strategies aiming to reduce mortality and improve quality of life [4].

In 2003, on available evidence the Council of the European Union recommended introduction of organized screening for breast cancer as one of the three cancer screenings [5]. While the main aim of organized breast cancer screening is early detection and reduction of breast cancer mortality [6]. A recent systematic review on mortality and breast cancer screening including evidence from randomized control trials and observational studies from across Europe has shown there is a significant reduction in mortality in women who were invited to screening versus women who were not (from 4% to 46% in different parts of Europe), and an even greater reduction in mortality in women who ever attend screening versus those who did not (from 2% to 89% reduction in different parts of Europe). Due to the lack of studies, no study from Central or Eastern Europe was included in the systematic review [7]. Similar patterns are observed with survival analysis, and again no studies from Central/Eastern part of Europe have been found to be included in the systematic review on social disparities in survival from breast cancer by Minicozzi [8].

Slovenia started introducing organized population-based screening for breast cancer in 2008. Prior to organized screening, opportunistic screening was available for women who were considered to be at a higher risk of developing breast cancer [9, 10]. The estimated participation rate in opportunistic screening was 14–21% [11]. With the introduction of organized screening, opportunistic screening is not available any more for women eligible for the organized screening programme. The organized screening is performed according to international recommendations–with mammography scans every two years, women aged 50–69 years without a history of breast cancer are invited to participate in the screening [12–14]. The screening programme is centrally organized and coordinated at the Institute of Oncology Ljubljana. Screening mammographies are performed at 18 locations (all of them have to apply the same quality standards which are regularly monitored), the reading of mammograms is central, with double reading procedure and consensus conferences in place. Diagnostic and treatment procedures for all women involved in screening are carried out in two hospitals (since 2018 besides Institute of Oncology, also University Medical Centre Maribor). All screening procedures are evaluated according to European Quality Assurance Guidelines [15]. Screening and all procedures for diagnosing and treating cancer are covered by obligatory health insurance in Slovenia [13] (according to Health Insurance Institute data [16], 99.6% of population is insured). The participation rate in breast cancer screening programme is high, in 2018 it was 74% [14]. The roll-out of the national screening programme was gradual, similar to other countries [17, 18]. In 2008, the Slovenian organised screening covered 14.4% of all eligible women, in 2013 33.3%, in 2015 46.6%, in 2016 72.5% and reached full coverage in early 2018 [12–14].

The roll-out is described in more details in [12]. The mayor bust in rolling-out was after 2015, when new strategic plan was adopted, with sufficient political and financial support. The roll-out was carried out in the capital and surrounding areas first, next expanding to the second largest city in Slovenia (Maribor), followed by other areas. Using the Eurostat definition of urbanization level in Slovenia [19], the roll-out from 2008–2010 included 70% of densely populated areas, 10% of intermediately populated areas and 5% of sparsely populated areas in Slovenia, and additionally including 62% of intermediately populated areas and 70% of sparsely populated areas only in the period 2016–2018. Since the roll-out period for the screening programme in Slovenia was gradual, people from the regions that were not yet included often expressed their dissatisfaction and also concerns about discrimination and unequal possibilities [eg. 20, 21], where their concerns were also in line with the already known overall worse health outcomes in the areas with lower socio-economic status [22], which are mostly also the areas with a lower urbanization level.

In Slovenia, an association between socio-economic status and cancer incidence has been documented [23], and more recently the association between socio-economic status (measured as European Deprivation Index–Slovene version) and survival/mortality of cancer patients was explored. We found that several cancers, including breast, demonstrate worse outcomes in the lower socio-economic groups [24]. Before 2008, we found no significant differences in breast cancer in stage at diagnosis with respect to the urbanisation level of the area where the patient was living at the time of diagnosis. Until now, we have not explored possible disparities brought about by unequal access to healthcare services.

The step-wise introduction of breast cancer screening programme in Slovenia offers a unique opportunity for this type of an epidemiological study.

The main objective of our study was thus to investigate whether the implementation of organized screening for breast cancer in Slovenia introduced inequalities due to unequal access to screening services. We aim to explore whether the step-wise implementation of organized screening has induced disparities in intermediate level outcomes (earlier diagnosis and treatment) in women diagnosed with breast cancer in the target period and consequently whether the survival in patients who were not included in organized screening or did not participate is lower.

## 2 Methods

Data from the Slovenian Cancer Registry (SCR) and the Registry of Organized screening for breast cancer (Screening Registry) were used in our analysis. The SCR is a population-based registry, which has been operating since 1950. Its quality and completeness are regularly monitored and it has been reaching the highest international standards since its foundation [2]. The Screening Registry includes data on all women in Slovenia who are eligible for breast cancer screening (aged from 50 to 69 years and having obligatory health insurance), their responses to invitations as well as mammography results and results of any further diagnostics. Linking both databases is possible since both include the Personal Identification Number which is a 13-digits unique personal identifier used in Slovenia. The linkage between the two registries has a legal basis in the Slovenian Healthcare Database Act [25].

**Fig 1:**
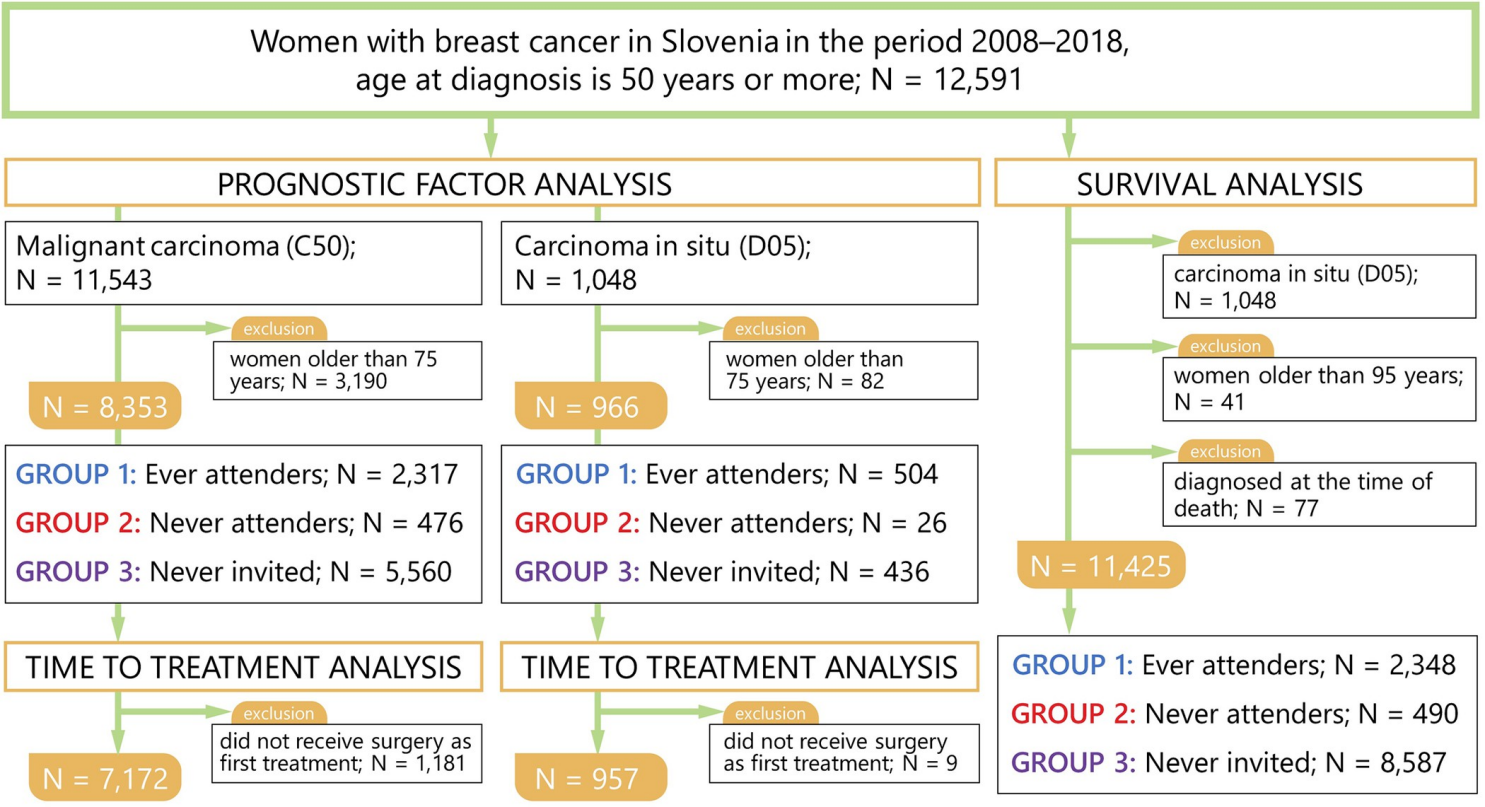
Inclusion and exclusion criteria. N = number of cases. ^1^ At least one mammography screening in organized screeningn programme prior to diagnosis. ^2^ At least one invitation to screening programme, but no screening performed. ^3^ No history of invitation or mammography within the screening programme.

The research study group included all female cancer cases coded as C50 –malignant neoplasm of the breast and D05 –carcinoma in situ of breast (according to the International Classification of Diseases, 10th edition)–registered in the SCR and diagnosed in the period 2008 to 2018 (Fig 1), their survival was observed until 1^st^ July 2021. The study group consisted of 12,591 women who were at diagnosis of age 50 or older. The data on the date of cancer diagnosis, age at diagnosis, stage at diagnosis (a simplified definition of stages at diagnosis is used, classifying them into in situ, localized, regional and distant stage of disease which is available in population-based cancer registries), type of primary treatment (surgery, systemic therapy, radiotherapy), starting date of cancer treatment and vital status at the end of follow up date (either date of death, date of lost to follow-up or date of the end of this study) were extracted from the SCR on the 1^st^ July 2021, with the vital status last updated on that day.

From the Screening Registry, we obtained the date(s) of invitation(s) and the date(s) of mammography(ies) performed within the screening program for the women in the study group. The data from the Screening Registry were extracted on 4^th^ January 2022. Information from the Screening Registry was used to create a new variable on screening programme status with three categories: (1) women who underwent at least one mammography within the screening programme any time before the diagnosis–ever attenders (i.e. diagnosed within as well as outside of the organized screening), (2) women who received at least one invitation to the screening programme, but have not attended–never attenders and (3) women who did not receive any invitation–never invited. We carried out the analysis in two ways: (A) comparing all three groups, because there is ample evidence showing that women who do not participate in screening programmes (group 2 or never attenders) differ substantially from the women who do [26–29] reflecting in worse outcomes and (B) comparing women who were included in the screening programme (group 1 and group 2 –invited) with women, who were not included in the screening programme (group 3 –not invited).

The data on the date(s) of invitation(s) and date(s) of mammography(ies) from the Screening Registry were compared with the dates of cancer diagnosis in SCR. Women who underwent a mammography within the screening programme after the diagnosis of breast cancer (8 women) and 382 of those who received the first invitation to screening after they were diagnosed with breast cancer were included in group 3 as their invitations/mammographies were actually systemic mistakes and they obviously had no benefit from being included in the screening programme.

In the prognostic factor analysis, we included only women who were 50–74 years of age at the time of diagnosis. The analysis was performed on 9,319 women (Fig 1). Though the Slovenian national organised screening programme invites women in the age group 50–69 years, the overall effect of the programme (notably due to lead time influencing the stage distribution of cancer discovered later and improving health awareness and health literacy among the invited) most likely extends further than age 69, that is why we are showing results for women aged 50–74 years at the time of diagnosis. We compared the stage of the disease at diagnosis among groups of women according to their screening programme status. The comparison of groups was performed with the χ^2^ test. Further, the time from establishing a diagnosis (with fine or core needle aspiration) to surgical treatment (in days) was analysed, also by stage. Here, only 8,129 women (7,172 women with C50 and 957 women with D05) who had surgery as their first treatment were included (Fig 1). We analysed the mean time to surgery with either ANOVA with the Bonferroni post hoc test (comparison of three groups in analysis A) or the t-test (comparison of two groups in analysis B). The analysis of prognostic factors was performed using IBM SPSS Statistics Version 22 software, p value of <0.05 was regarded as significant.

In the survival analysis we applied the net survival method by Pohar-Perme [30]. The net survival is the survival that would be observed if the only cause of death was from the disease under study (breast cancer in our case). To diminish the lead time bias we present the five-year net survival with their 95% confidence intervals (CI) by stage of the disease. There were 11,425 women included in the survival analysis; women above 95 years of age (N = 41), women who had their diagnosis determined on the day of death (N = 77) and women who had carcinoma in situ (N = 1,048) were excluded (Fig 1). Not all women included in the analysis were followed-up for five years, therefore the complete approach was used in the survival calculations [31]. The last date of the vital status update was the 1^st^ July 2021. For calculations, we used the relsurv package version 2.2–6 for the R software version 4.1.0 [32].

Research, using data from Slovenian Cancer Registry or Breast Cancer Registry, which both have legal background for collection, linkage and use of these data, does not require ethical approval and/or participant consent.

## 3 Results

We performed analysis of malignant neoplasms (C50–8,353 women) and carcinoma in situ (D05–966 women).

**Fig 2:**
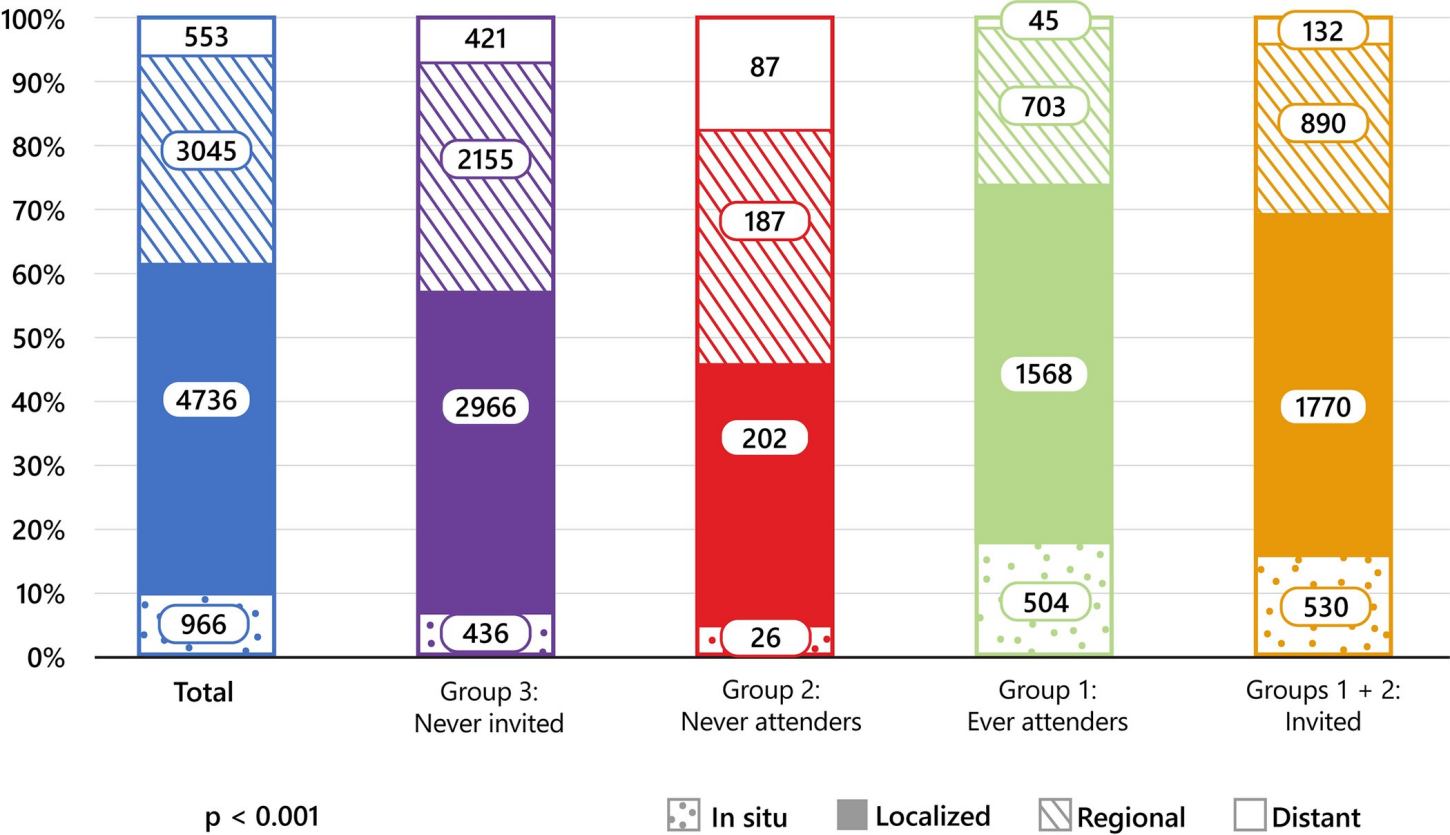
Number and proportion of women with breast cancer aged 50–74 years by screening programme status and stage at diagnosis, Slovenia, 2008–2018. ^1^ At least one mammography screening in organized screening programme prior to diagnosis. ^2^ At least one invitation to screening programme, but no screening performed. ^3^ No history of invitation or mammography within the screening programme.

In the prognostic factors analysis there were 9,319 women in total included in the stage analysis and 8,129 in the subsequent time-to-first-surgical-treatment analysis. Two thirds of patients (5,996 cases) included in the stage analysis were never invited to screening (group 3), 5% (502 women) were never attenders (group 2) and the rest (2,821 patients; 30%) were ever attenders (group 1). There was a statistically significant difference in the stage of disease at the time of diagnosis among the groups (Fig 2), where ever attenders (group 1) had the highest proportion of cancers discovered in the in situ stage: 17.8% vs. 7.2% in never invited (group 3) and 5.1% in never attenders (group 2), similarly they had the highest proportion of localized stage: 55.6% vs. 49.5% in never invited (group 3) and 40.2% in never attenders (group 2). Never attenders (group 2), had the highest proportion of cancers at the time of diagnosis in distant stage: 17.3% vs. 7.0% in never invited (group 3) and only 1.6% in ever attenders (group 1).

In the analysis B where the effect of screening availability was examined there were 3,323 (35%) women included in the group invited to screening before the diagnosis. Similarly, to the analysis A, these comparisons show statistically significant differences in stage distribution (all p<0.001), where women invited to the screening programme had greater proportion of cancer cases diagnosed with in situ carcinoma (15.9%) compared to 7.2% in women never invited. The same was true for women who were diagnosed with localized stage (53.3%) compared to 49.5% in women never invited to screening, the opposite was true for distant stage of the disease (3.9% invited to screening vs. 7.0% never invited to screening) (Fig 2). Distribution of stages before the introduction of organized screening (period 1998–2007) was similar to distribution of stages in women never invited to screening (group 3) in our analysis, that is 6.0% of in situ cancers, 47.5% of localized stage, 36.0% of regional and 8.4% of distant stage of the disease at the time of diagnosis.

For the analysis of the time from diagnosis to surgery as first treatment (Table 1) we calculated the mean time for each stage of the disease and compared women with different screening programme statuses.

**Table 1:** Time from diagnosis of breast cancer to surgery as first treatment (number of cases in each group (N) and mean time in days with standard deviation (SD)) in women aged 50–74 years, by stage at diagnosis and screening programme status, Slovenia, 2008–2018.

	Total	Group 1	Group 2	Group 3	p
	N (%)	Ever attenders^1^	Never attenders^2^	Never invited^3^	
		N (%)	N (%)	N (%)	
MALIGNANT CARCINOMA (C50)—ALL STAGES TOGETHER					
N^a^	7,172	2,156	312	4,704	
Mean time in days (SD)	37.8 (26.9)	40.3 (17.6)	47.1 (32.5)	36.0 (29.6)	<0.001
LOCALIZED					
N	4,530	1,532	177	2,821	
Mean time in days (SD)	38.0 (26.2)	40.4 (17.8)	47.2 (31.7)	36.1 (29.3)	<0.001
REGIONAL					
N	2,540	614	124	1,802	
Mean time in days (SD)	37.4 (26.5)	40.0 (16.9)	45.4 (29.2)	36.0 (28.7)	<0.001
DISTANT					
N	100	10	11	79	
Mean time in days (SD)	36.3 (52.7)	41.0 (20.7)	64.1 (65.1)	31.8 (52.9)	0.156
CARCINOMA IN SITU (D05)					
N	957	500	25	432	<0.001
Mean time in days (SD)	47.4 (33.4)	43.2 (22.0)	76.1 (31.2)	51.2 (42.5)	

^a^ There were 2 cases of C50 that did not have stage definition–not shown.

^1^ At least one mammography screening in organized screeningn programme prior to diagnosis.

^2^ At least one invitation to screening programme, but no screening performed.

^3^ No history of invitation or mammography within the screening programme.

For the malignant neoplasms (C50) the mean time to surgery as first treatment ranged from 36 days to 47 days in the groups with different screening status with the longest mean time in the group of never attenders (group 2). Looking at different stages of the disease in all screening status groups, the mean time to surgery was shortest in all stages of the disease in the group never invited to screening (group 3). The mean time was shortest for distant stage at diagnosis (36.3 days; standard deviation (SD) 52.7 days). In ever attenders (group 1), the mean time to surgery was very similar in all stages and was around 40 days. Even though it was longer than in the group of women never invited (group 3), the mean time in ever attenders (group 1) had a smaller deviation despite lower group numbers (SD from 17 to 20.6 days for different stages of the disease, while the SD for women never invited (group 3) ranged from 28.7 to 52.9 days for different stages of the disease). In all stages of the disease, the mean time to surgery was longest in the group of never attenders (group 2).

For the carcinoma in situ (D05) the mean time from diagnosis to surgery ranged from 43 days to 76 days and was on average 10 days longer compared to the mean time for the malignant form of the disease (C50). The longest mean time to surgery for carcinoma in situ was in never attenders (76.1 days) and the shortest in ever attenders (43.2 days).

In the analysis B, the mean time to surgery as first treatment in the malignant neoplasms (C50) ranged from 36 days to 41 days with longer mean time in the group invited to screening, but again showing much smaller variability, especially in the local and regional stage (SD 20 days in the group invited to screening vs. 29 days in the group never invited), while in the distant stage of the disease the difference between the two groups was much greater but not significant (Table 2).

**Table 2:** Time from diagnosis of breast cancer to surgery as first treatment (number of cases in each group (N) and mean time in days with standard deviation (SD)) in women aged 50–74 years, by stage at diagnosis and availability of organised screening programme, Slovenia, 2008–2018.

	Total	Never invited to screening programme	Invited to screening programme	p
MALIGNANT CARCINOMA (C50)—ALL STAGES TOGETHER				
N^a^	7,172	4,704	2,468	
Mean time in days (SD)	37.8 (26.9)	36.0 (29.6)	41.2 (20.2)	<0.001
LOCALIZED				
N	4,530	2,821	1,709	
Mean time in days (SD)	38.0 (26.2)	36.1 (29.3)	41.1 (19.8)	<0.001
REGIONAL				
N	2,540	1,802	738	
Mean time in days (SD)	37.4 (26.5)	36.0 (28.7)	40.9 (19.6)	<0.001
DISTANT				
N	100	79	21	
Mean time in days (SD)	36.3 (52.7)	31.8 (52.9)	53.1 (49.5)	0.537
CARCINOMA IN SITU (D05)				
N	957	432	525	<0.001
Mean time in days (SD)	47.4 (33.4)	51.2 (42.5)	44.4 (23.0)	

^a^ There were 2 cases that did not have stage definition–not shown.

**Fig 3:**
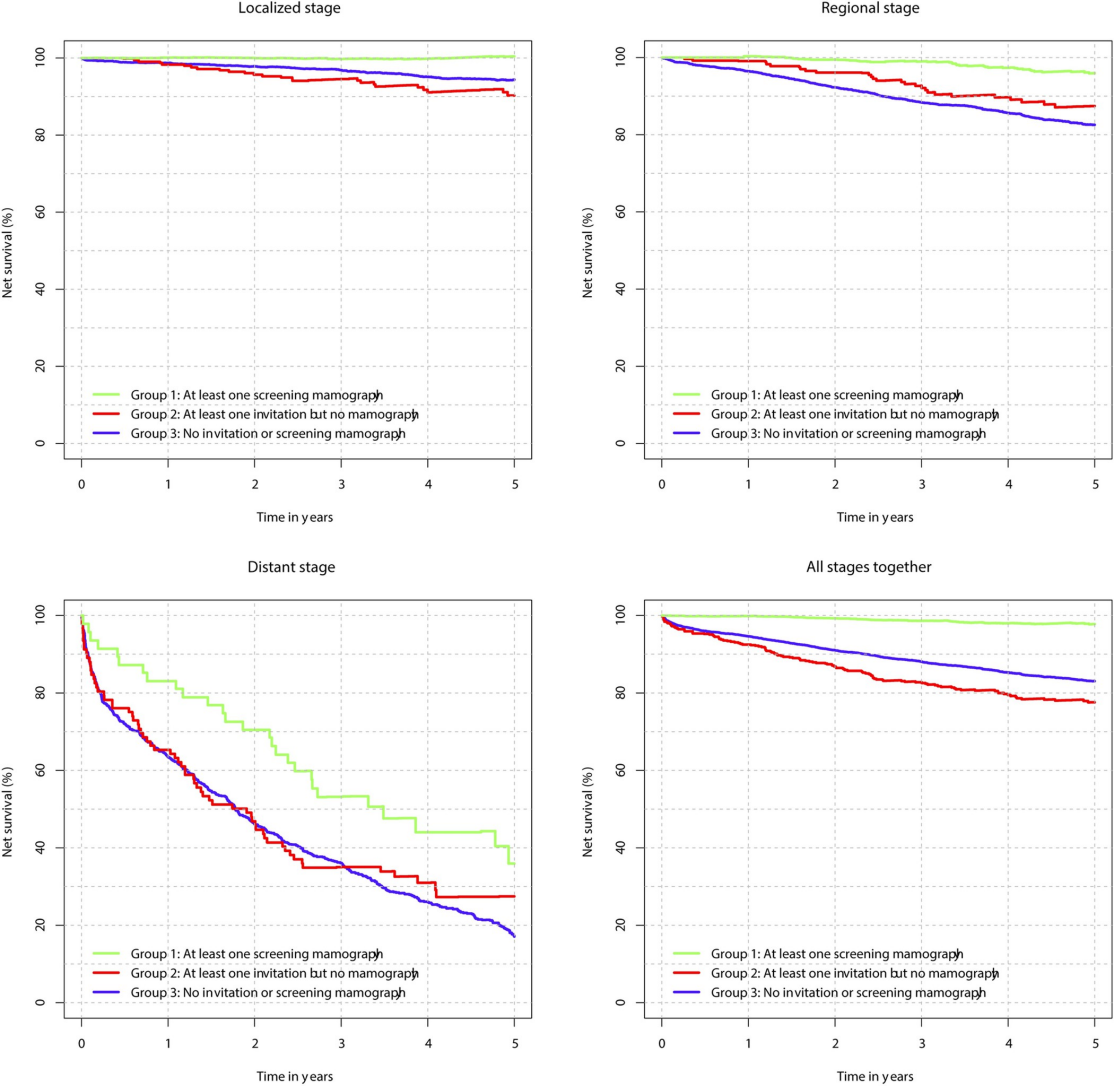
Five-year net survival for women with breast cancer according to stage and screening program status, Slovenia 2008–2018.

Survival analysis showed the highest net survival for ever attenders (group 1). That was true for all stages of the disease. In the local stage, as well as in the regional stage, the 5-year net survival of ever attenders (group 1) (local stage: 100.4%; CI: 99.4–101.5%; regional stage: 96%; 95% CI 93.7–98.3%) was statistically significantly different from the other two groups (group 2 and 3), while the other two groups did not differ significantly (never invited (group 3): local stage: 94.4%; 95% CI 92.8–96.1%; regional stage: 82.6%; 95% CI: 80.6–84.6%; never attenders (group 2): local stage: 90.3%; 95% CI: 85.0–95.8%; regional stage: 87.4%; 95% CI 81.8–93.5%) (Fig 3). For the distant stage, the difference in 5-year net survival was significant only between ever attenders (35,9; 95% CI 22,9–56,3) and those never invited (17,1; 95% CI 14,0–20,8).

## 4 Discussion

To our knowledge this is one of the first studies in a Central European country on prognostic factors and outcomes of breast cancer patients from the population where organized breast cancer screening was available. The main aim of organized breast cancer screening programmes is early detection of cancer and reduction in mortality, which can be achieved through a systematic population-based approach with appropriate quality assurance at all levels [4, 6, 33]. Thus, one important aspect of systematic organized screening programmes is to reduce inequalities stemming from individual or territorial socio-economic characteristics through more equal access to screening services and treatment for all [33]. Due to the gradual roll-out of organized screening in Slovenia (11 years), we aimed to take advantage of this step-wise implementation in comparing women with and without access to organized screening, where we should keep in mind, that the roll-out was carried out first in the more densely populated areas, and covering the more rural areas at a later stage, where rural areas in Slovenia are also socio-economically worse off.

The results of our study on the stage of the disease among different groups of women according to their screening programme status shows the same results as several previous studies, namely that participation in organized screening enables the discovery of breast cancer at an earlier stage [4, 33]. We found that the proportion of localised stage of breast cancer and carcinoma in situ was the highest in ever attenders (group 1). The results of our study also show, that they have better long-term outcomes which we measured as survival. In general, the earlier a cancer is diagnosed the better the chances are for successful treatment, leading to longer survival [34]. This is true for most cancer sites, but when using survival analysis in relation to screening status, you have to bear in mind the possibility of lead time bias–bias that occurs when a disease is detected by screening at an earlier time point than it would have been if it had been diagnosed by its clinical appearance, but when with further treatment the effect on the outcome is minimal, thus only artificially prolonging the survival time–, which we tried to avoid by using survival analysis by stage.

As expected, the mere possibility of organized screening programme does not suffice for eradication of inequalities [35, 36]. Countries should therefore be aware of possible additional inequalities introduced during the roll-out period of national organized screenings. In Slovenia previous research [23, 24] has shown that groups with lower socio-economic position, who usually also live in more rural areas, have worse health outcomes. In our study we have shown that outcomes were worse for women who did not have the chance to participate in the screening programme (never invited (group 3)), since it was not yet available in their region. While the more rural areas of the country were included in screening later in the roll-out phase, we can say that the direction of screening implementation in Slovenia was incoherent with the pre-existing socio-economic inequalities. Roll-out of any national programme is usually gradual and should follow the capacities provided to ensure high quality of the screening programme, but the roll-out period should be as short as possible to avoid unnecessary additional inequalities imposed by unequal access to services. In our analysis we also presented great differences among ever attenders and never attenders. As is known from previous studies [26–29, 37, 38], non-attenders are a specific group of people, who are in general exposed to different risk factors, individual as well as environmental, including lower area-level socio-economic position. Specific components of behaviour in the never attenders group were not explored in our study, though the finding of time to surgery as first treatment being the longest in the group of never attenders for all stages of the disease, might imply that this group of women holds a different attitude also towards cancer treatment. As experts warn, tackling inequalities in access to and participation in cancer screening will also during full coverage of a screening programme likely require a more tailored approach to better address the needs and barriers for groups that are under-screened [33].

Our time to treatment analysis has revealed interesting findings. As expected, mean time to surgical treatment was longest in never attenders (group 2). It also showed that the mean time to surgical treatment was lower for women never invited to screening (group 3) (for 3–4 days), but the variability in time was much lower among ever attenders (group 1). In line with our results, a longer diagnosis to treatment interval in a specialized pathway for breast cancer care vs. regular pathway was found in Canada by Webber et al [39]. They assumed this was the consequence of either greater use of staging investigations or multidisciplinary consultations for treatment planning before initiation of treatment in the specialized pathway [39]. We assume that the latter could also be the case in Slovenia. We know that quality assurance protocols are in place in the organized screening since the beginning, with the time interval for further assessment defined (4–10 working days) and findings discussed at weekly preoperative multidisciplinary conferences, where decisions on further treatment are taken [12, 13, 40]. These protocols are not followed in all medical centres providing breast cancer diagnosis and treatment outside the organized screening programme (women in the never invited group were treated in 18 different health care facilities, only 26% of them in the hospital included in organized screening). Outside organized screening the usual reading of mammographies is done by one expert, the time interval for readings varies, the diagnostic procedures are not monitored to follow specific guidelines, also most of the smaller hospitals do not have multidisciplinary conferences prior to starting treatment [40], which could all reflect in suboptimal treatment and outcomes. Our hypothesis is also based on the fact that women who were never invited (group 3) had a higher proportion of diagnosis made on fine needle aspiration only (3.3% vs. 0.3% in ever attenders (group 1)–not show in results section), immediately followed by surgery without carrying out additional diagnostic procedures, which can significantly lower the time to surgery interval. In our analysis there were 17.3% of women who had surgery before day 10 after diagnosis in the group never invited in screening (group 3) vs. only 1.2% in the ever attenders group (group 1). In the included period ever attenders (group 1) were in 80% diagnosed in one hospital–Institute of Oncology Ljubljana, which was at the time the only hospital performing diagnostic and treatment procedures in the organized screening programme and has had multidisciplinary conferences for all women with breast cancer, regardless of their screening programme status; 80% of them were diagnosed actually within the screening programme). The more extensive diagnostic work-up and multidisciplinary approach could lead to prolonged mean time from diagnosis to treatment for women within the organized screening, while providing better quality of care in the long run. This hypothesis could reflect also by the results of our survival analysis. The international evidence whether longer time to treatment is associated with worse outcomes are conflicting. Some studies have found out that longer time to treatment is associated with worse survival, especially in the early stages of breast cancer [e.g. 41–43], other studies have found that longer time to treatment interval does not influence the survival or progression free survival if the interval is prolonged (within reasons) due to more extensive diagnostic work-up or for the sake of more suitable decision-making process [e.g. 44, 45].

We should bear in mind that comparison of survival by stage could also be influenced by variability in the process of stage recording [4]. In our case the variability could arise between hospitals, depending on the diagnostic procedures performed to determine stage, which could lead to underestimation of stages outside the screening programme and thus lead to worse survival for this group of women. It would be useful to have other data to better define the stage of the disease such as tumour diameter, number of lymph nodes involved etc., but unfortunately this kind of data is not available in population registries.

Despite the fact that time to treatment in the group of ever attenders (group 1) in the observed period has not yet reached the target value set by the European standards for quality of care [15], our survival analysis affirms that management of patients included in the organized screening programme provides better outcomes. The financial accessibility of healthcare outside organized screening could not play an important role since in Slovenia universal health insurance covers all expenses of cancer treatment [46]. Similar findings are present all over Europe, while also exhibiting large variations between countries and within countries [8]. Unfortunately, we do not have the data on the time interval from mammography to diagnosis for all women with breast cancer. From clinical experiences from our Institute and from other studies [e. g. 39], this interval is much longer for women not included in organized screening, which also partially explains worse stages of cancers in women outside screening. From our results, we can assume that women not included in the screening programme were treated for breast cancer, but their treatment was suboptimal.

Unfortunately, currently data in Slovenia does not enable us to further explore the quality of treatment at different locations, since data on treatment in population-based cancer registries is limited. Thus, we use proxy indicators such as survival, which is a key indicator of the overall effectiveness of the health system in a country, reflecting both individual characteristics as well as characteristics of the health care system [8, 47]. We are looking forward to exploring the issue of quality of treatment in the future, since SCR is in the process of establishing a national clinical registry for breast cancer patients, where a much richer set of data on diagnostic and treatment procedures will be available.

In our study we did not explore whether there is a reduction in mortality attributable to the implementation of organized breast cancer screening in Slovenia, which would also serve as monitoring the efficiency of the screening programme. This remains one of our future challenges and because mortality due to breast cancer is low, we expect to have enough cases for relevant analysis in a few years’ time.

### 4.1 Strengths and limitations

The biggest strength of our study lies in the fact that we used high quality data covering the whole population, both on breast cancer patients as well as regarding organized screening. Thus, we can evaluate the effect of stepwise implementation of screening on the whole population, as different parts of the population were covered at different times. The highly accurate and complete data of the Screening Registry allows for correct classification of women according to their organized screening status, i.e. whether (and when) they were invited and whether (and when) they underwent a mammography screen. In the analysis we covered a period of 11 years, providing us with sufficient cases despite the relatively small population of Slovenia.

The biggest limitation is the lack of individual data on opportunistic screening. We have an estimate on participation rate at opportunistic screening from the period 1998–2002, which is 14–21% [11]. This participation rate could have differed substantially between regions, since participation was highly dependent on professional advice from personal gynaecologist working at primary level of healthcare. Lack of such individual data diminishes the differences between the group that was included in organized screening and the one that was not, since women from the group which was not included could have been undergoing opportunistic mammography screening.

We also lack the data for the symptomatic women or for women undergoing opportunistic screening on time interval from the first contact with the health system to diagnosis by fine needle or core aspiration. The time interval needed to establish a diagnosis would give us insights into other aspects of our health system which can also potentially influence outcomes. Part of this data will be available through the establishment of a clinical breast cancer registry, which is already taking place in Slovenia and will include data from 2022 on.

Our analysis would also bring additional insights if we could also include some kind of (individual) socio-economic indicator as explanatory variable. Unfortunately, at this stage this data was not yet available, but linking of data in our analytical database with a small area level socio-economic indicator (Slovene version of the European Disparity Index [48]) is foreseen.

We realise that when using survival in relation to organized screening programme you have to consider lead time bias–bias introduced by discovering the disease at an earlier (asymptomatic) stage than without screening, but not adding anything to better survival, only prolonging the time with known diagnosis. We tried to address this bias by performing survival analysis by stage of cancer, where more similar cases of disease are present and assuming that the lead time bias is the smallest in the earlier stages, though we cannot rule out residual bias.

We know that there is also a slight selection bias in the screening group towards older women. This is because when screening was introduced in a certain area, per consensus the first women to be invited were those who were just below the upper age limit criteria, since they were the first to “fall out” of the screening eligibility criteria in the years to come. To a certain extent this selection bias was attenuated by analysing a long (eleven year) period of organized screening operation, which means that in the second and further years of operation in a given area, all eligible women were invited to screening.

Finally, we have to acknowledge the fact, that our analysis does not fully capture the influence of organized screening on the population, since it includes a relatively short follow-up period since complete roll-out. Our observation period for survival analysis is until mid 2021, which means that only women who attended screening mammography in the year 2015 had the potential for the whole 5-year follow up. In the year 2015 less than half of eligible women were invited to organized screening. For assessment of full impact of organized screening on a population, at least 15 to 20 years of follow-up after complete roll-out is necessary [5].

## 5 Conclusion

Our study is one of the first studies in a Central European country on prognostic factors and outcomes of breast cancer patients from the population where organized breast cancer screening was available. This was possible due to the availability of high-quality data from the screening and population-based cancer registries. We explored prognostic factors and outcomes in groups of women according to their screening programme status throughout the eleven-year roll-out period which gave us the opportunity to observe results of a step-wise implementation with potential for undesired social effects.

The results showed that women with the possibility to participate in the screening programme had cancers diagnosed at earlier stages. Despite the fact that their time to first surgical treatment was longer than in the group of women who were not part of the screening programme, their survival rates by stage were higher. This implies that screening programmes with their quality assurance protocols play an important role in improving long-term outcomes. Therefore, all countries implementing organized screening should try to minimize the roll-out period in order to avoid temporary inequity in access to care. In the future, the benefits that come about as the result of the screening programme should expand beyond the programme to include improving equity in the society.
